# Supplementary material for: Precise control of alloying sites of bimetallic nanoclusters via surface motif exchange reaction
Source: Nat Commun. 2017 Nov 16;8:1555. doi: 10.1038/s41467-017-01736-5 (PMC5691201; doi:10.1038/s41467-017-01736-5)
Supplement: Supplementary file 1 — Supplementary Information [file 41467_2017_1736_MOESM1_ESM.pdf]

Supplementary Note 1. Characterization of as-synthesized $[Ag_{44}(SR)_{30}]^{4-}$ nanoclusters (NCs).

UV-vis absorption spectrum of the as-prepared Ag NCs shows several characteristic absorption features of the reported $[Ag_{44}(SR)_{30}]^{4-}$ NCs (SR denotes thiolate ligand) at 415, 485, 535, 645, and 835 nm (Fig. 1a)[1], which also suggests good yield (~90% on Ag atom basis, determined by inductively coupled plasma optical emission spectroscopy, ICP-OES) and high purity of the as-prepared $[Ag_{44}(p\text{-MBA})_{30}]^{4-}$ in our protocol. To further assess the purity of as-prepared $[Ag_{44}(p\text{-MBA})_{30}]^{4-}$ NCs, we analyzed our samples by polyacrylamide gel electrophoresis (PAGE) and electrospray ionization mass spectrometry (ESI-MS). As shown in Fig. 1a (left inset), the PAGE result shows 4 bands, where the most prominent band (Band 4) corresponds to $[Ag_{44}(p\text{-MBA})_{30}]^{4-}$, and the other three bands (Bands 1-3) could be attributed to the impurities generated during the running of PAGE analysis, which is consistent with a previous report[2]. ESI-MS spectrum (Fig. 1b) shows two dominant peaks at *m/z* of ~2335 and ~2975 in a broad *m/z* range of 1000–4000, which should be assigned to $[Ag_{44}(p\text{-MBA})_{30}]^{4-}$ cluster ion and a fragment ion of $[Ag_{43}(p\text{-MBA})_{28}]^{3-}$, respectively. The good accuracy of our assignment can be exemplified by the perfect match of the experimental and calculated isotope patterns of $[Ag_{44}(p\text{-MBA})_{30}]^{4-}$ (Fig. 1b, left inset). It should be noted that our PAGE results and ESI-MS spectrum are identical to those of the reported pure $[Ag_{44}(p\text{-MBA})_{30}]^{4-}$ NCs[2].

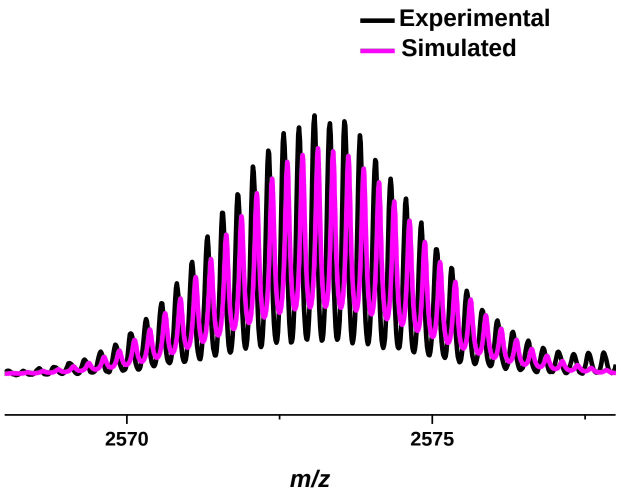

Supplementary Figure 1. Experimental (black line) and simulated (magenta line) isotope patterns of $[Ag_{32}Au_{12}(SR)_{29}Cl]^{4-}$, where SR denotes thiolate ligand.

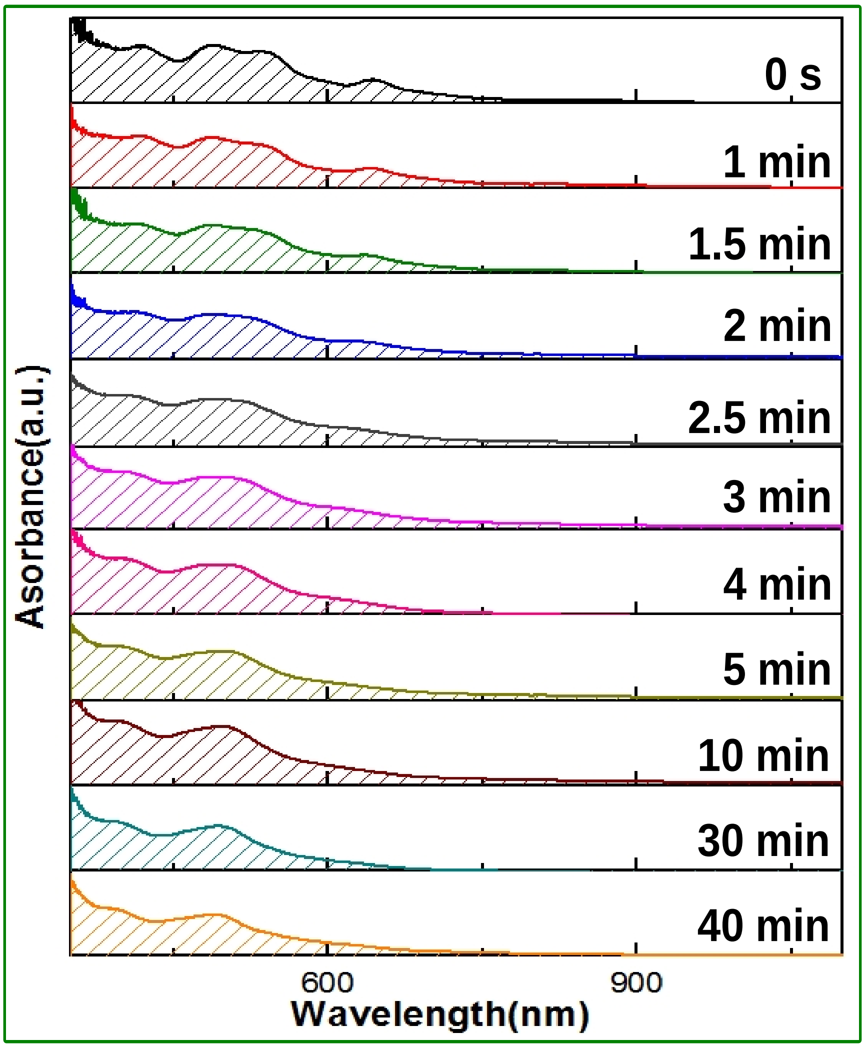

Supplementary Figure 2. Time-evolution ultraviolet-visible absorption spectra of reaction mixture from $[Ag_{44}(SR)_{30}]^{4-}$ to $[Ag_{32}Au_{12}L_{30}]^{4-}$ nanoclusters (L = SR or Cl).

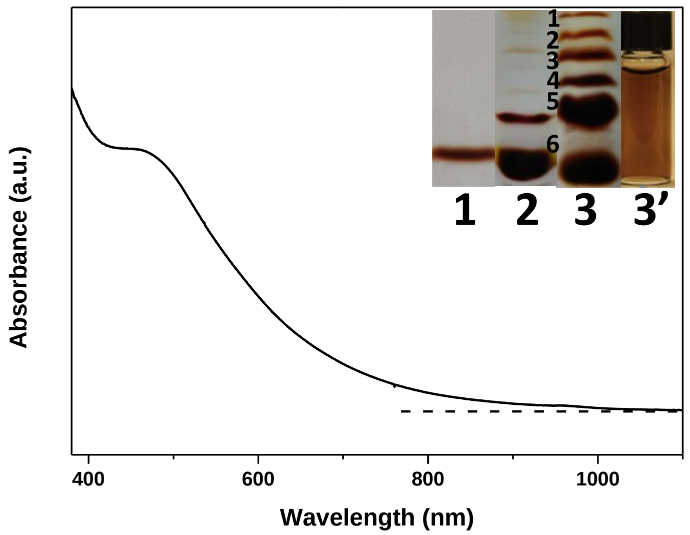

Supplementary Figure 3. Ultraviolet-visible absorption spectrum of AgAu nanoclusters formed by reacting $[Ag_{44}(SR)_{30}]^{4-}$ with Au(III) salts (i.e., $HAuCl_4$). The zero absorbance is indicated by the dotted line. Insets 3 and 3' are polyacrylamide gel electrophoresis result and digital photo of the as-formed AgAu nanoclusters. For comparison purpose, the polyacrylamide gel electrophoresis results of $[Ag_{32}Au_{12}L_{30}]^{4-}$ and $[Ag_{44}(SR)_{30}]^{4-}$ are shown as insets 1 and 2, respectively.

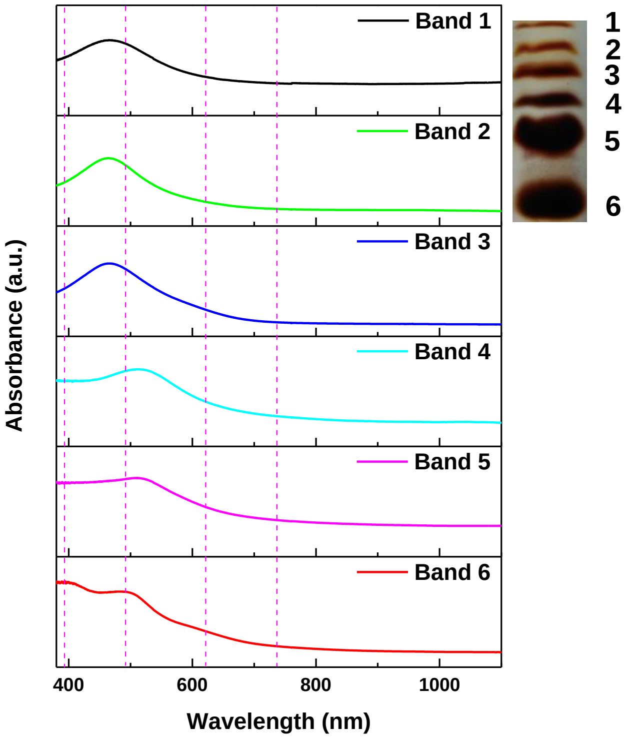

Supplementary Figure 4. Ultraviolet-visible absorption spectra of Bands 1–6 identified in the polyacrylamide gel electrophoresis gel (inset) of cluster mixture formed by reacting $[Ag_{44}(SR)_{30}]^{4-}$ with $HAuCl_4$. The dotted lines indicate the absorption features of $[Ag_{32}Au_{12}L_{30}]^{4-}$ at 390, 490, 620, and 735 nm.

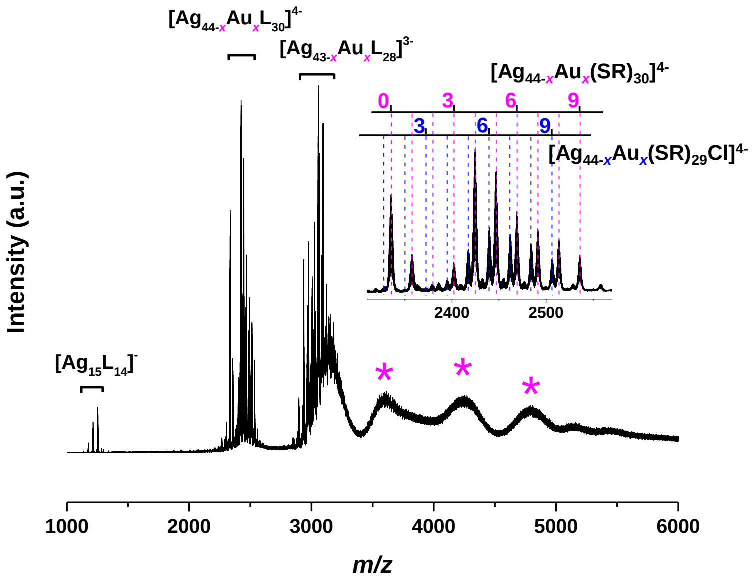

Supplementary Figure 5. Electrospray ionization mass spectrum of AgAu nanoclusters formed by reacting $[Ag_{44}(SR)_{30}]^{4-}$ with Au(III) salts (i.e., $HAuCl_4$). The inset shows zoom-in spectrum of $[Ag_{44\text{-}x}Au_xL_{30}]^{4-}$ (L = SR or Cl) peaks. The $[Ag_{43\text{-}x}Au_xL_{28}]^{3-}$ is a common fragment of $[Ag_{44\text{-}x}Au_xL_{30}]^{4-}$, similar to the fragment ion of $[Ag_{43}L_{28}]^{4-}$ observed in mass spectrum of $[Ag_{44}L_{30}]^{4-}$ (Fig. 1b in main text). The asterisk peaks correspond to nanoclusters with larger sizes, whose accurate formula could not be deduced due to a lack of isotope resolution.

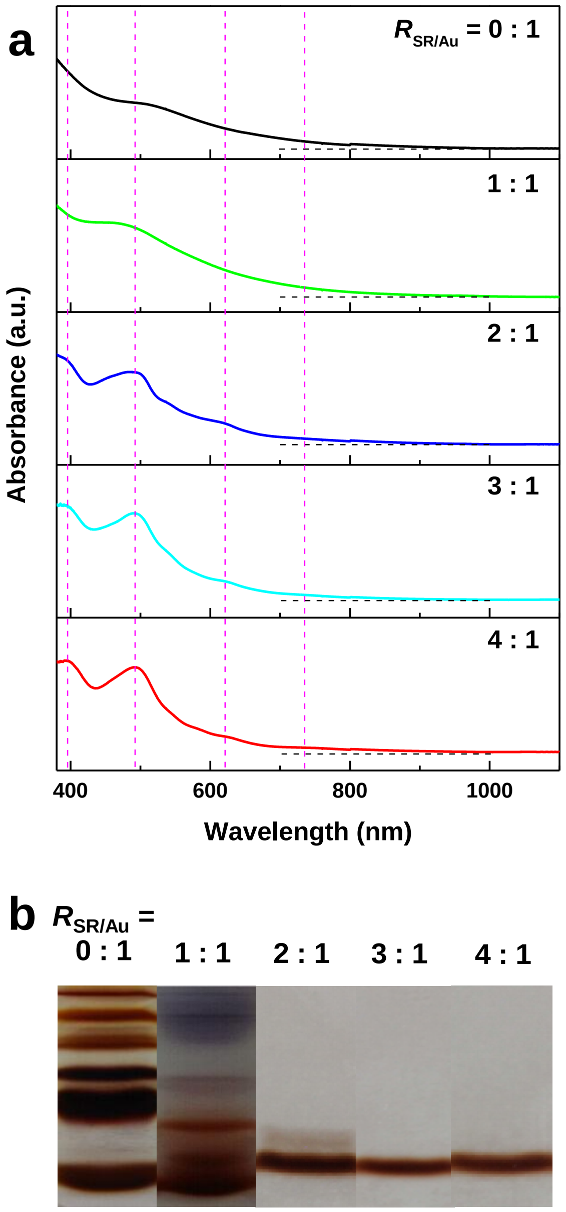

Supplementary Figure 6. (**a**) Ultraviolet-visible absorption spectra and (**b**) polyacrylamide gel electrophoresis results of AgAu nanoclusters formed by reacting $[Ag_{44}(SR)_{30}]^{4-}$ with Au(I)-SR complexes prepared by varied thiol-to-Au ratios, $R_{SR/Au}$. The dotted drop lines in (**a**) serve as visual guide for characteristic absorptions of $[Ag_{32}Au_{12}L_{30}]^{4-}$ NCs.

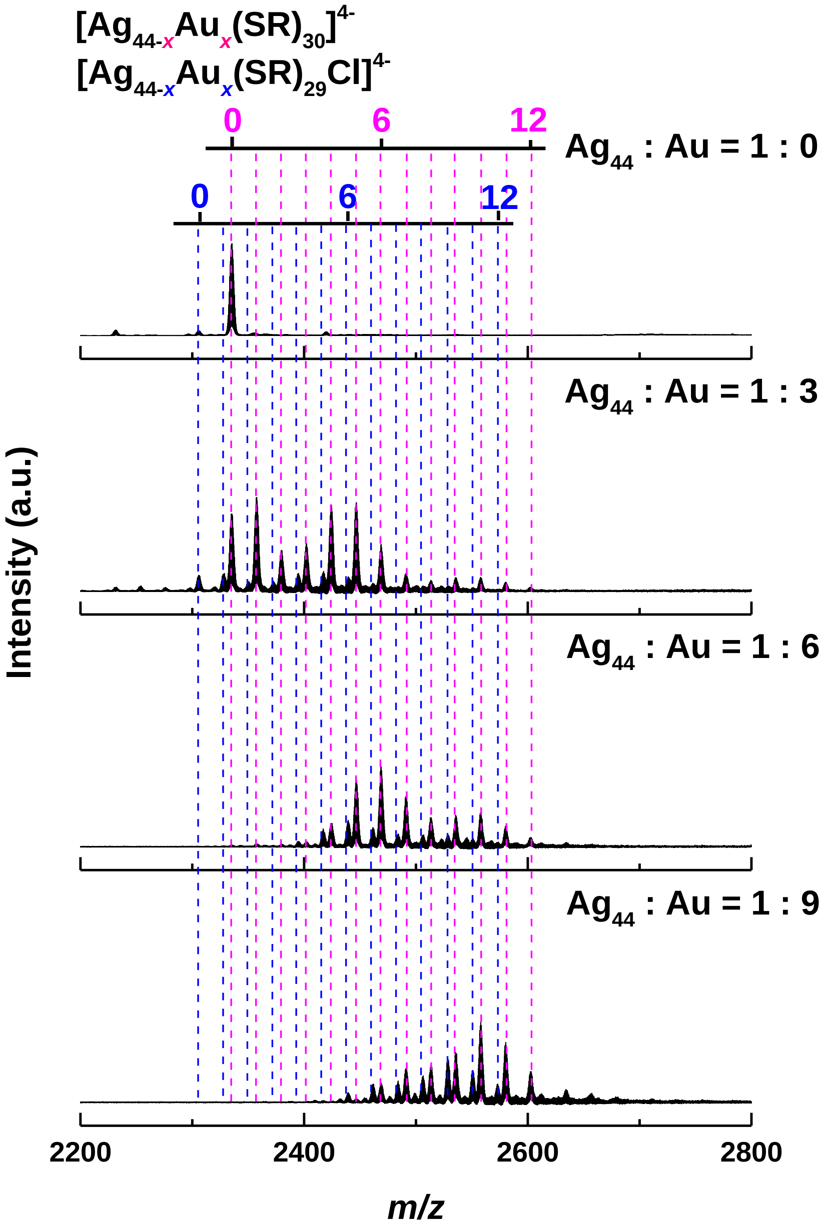

Supplementary Figure 7. Zoom-in electrospray ionization mass spectra of $[Ag_{44-x}Au_xL_{30}]^{4-}$ NCs synthesized at varied $R_{Ag44/Au(I)}$ ranging from 1:0 to 1:9.

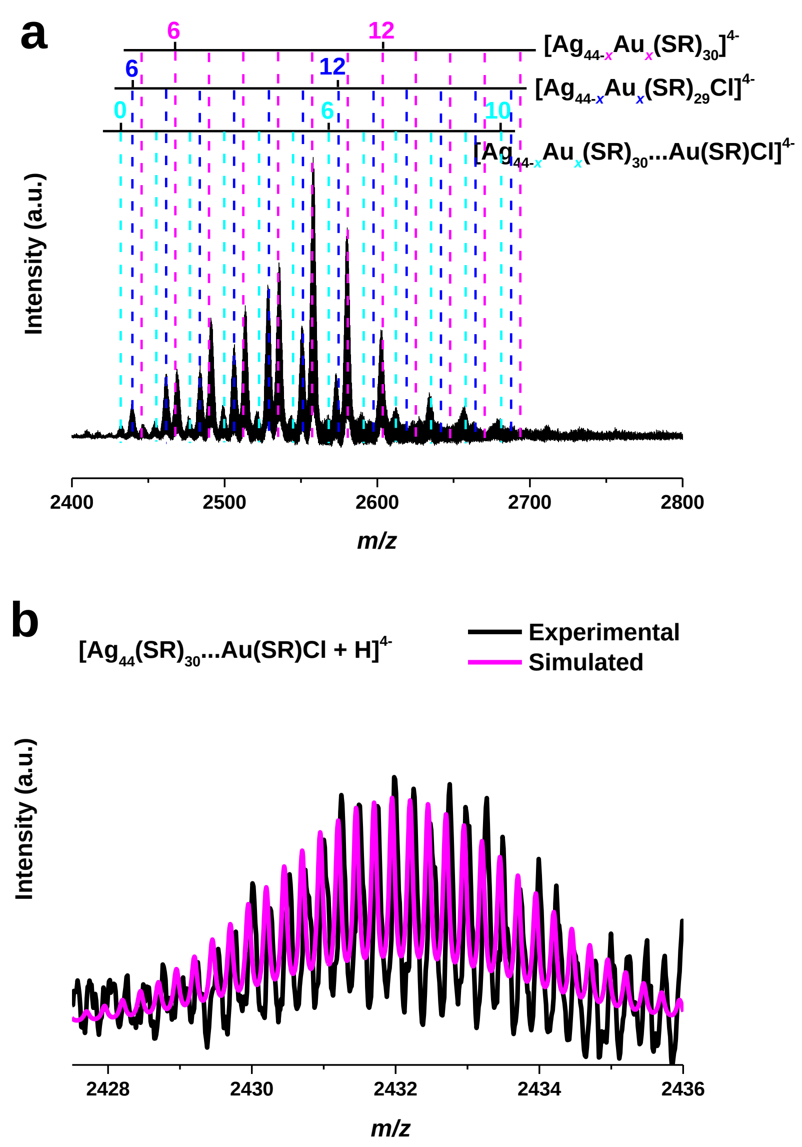

Supplementary Figure 8. (**a**) Zoom-in electrospray ionization mass spectrum of $[Ag_{44-x}Au_xL_{30}]^{4-}$ nanoclusters synthesized at $R_{Ag44/Au(I)}$ = 1:9, where peaks with mass higher than that of $[Ag_{32}Au_{12}(SR)_{30}]^{4-}$ could be attributed to $[Au(SR)Cl]^-$-associated $[Ag_{44-x}Au_x(SR)_{30}]^{4-}$ (x = 0-10; $[Ag_{44-x}Au_x(SR)_{30}\cdots Au(SR)Cl + H]^{4-}$). (**b**) Experimental (black line) and simulated (magenta line) isotope patterns of $[Ag_{44}(SR)_{30}\cdots Au(SR)Cl + H]^{4-}$.

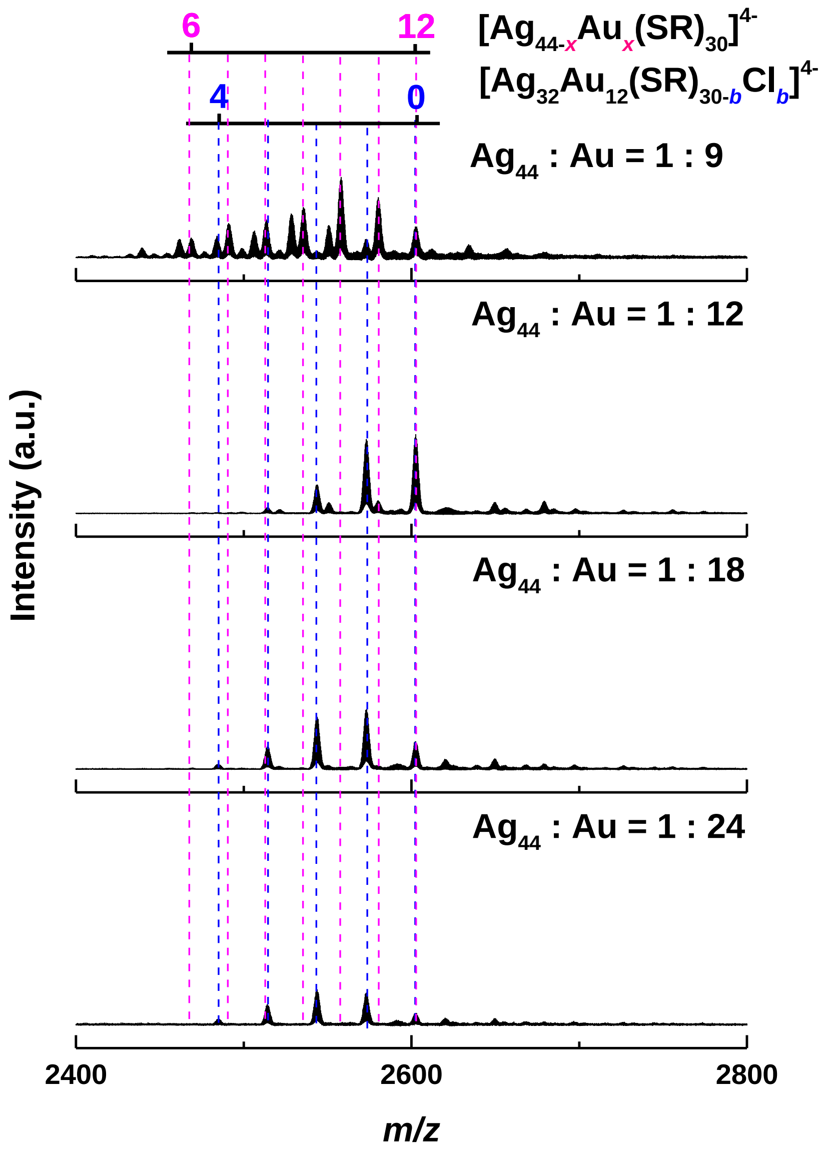

Supplementary Figure 9. Zoom-in electrospray ionization mass spectra of $[Ag_{44-x}Au_xL_{30}]^{4-}$ nanoclusters synthesized at varied $R_{\mathrm{Ag44/Au(I)}}$ ranging from 1:9 to 1:24.

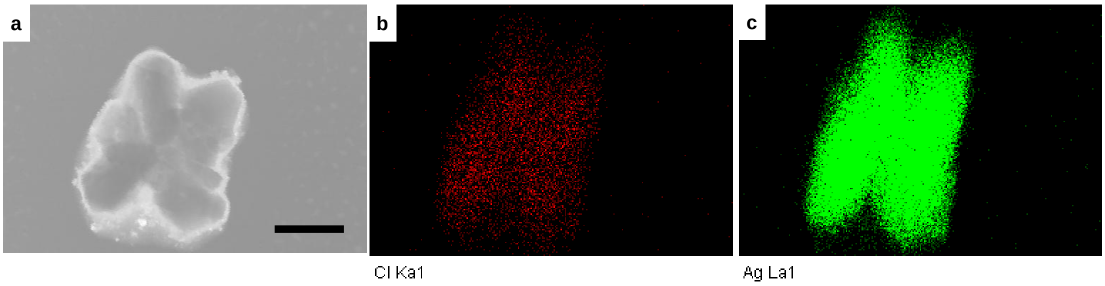

Supplementary Figure 10. (**a**) Scanning transmission electron microscopy (STEM) image and (**b**, **c**) energy dispersive X-ray (EDX) elemental maps in terms of (**b**) Cl and (**c**) Ag of the by-product AgCl formed in the alloying reaction. The scale bar is 10 nm.

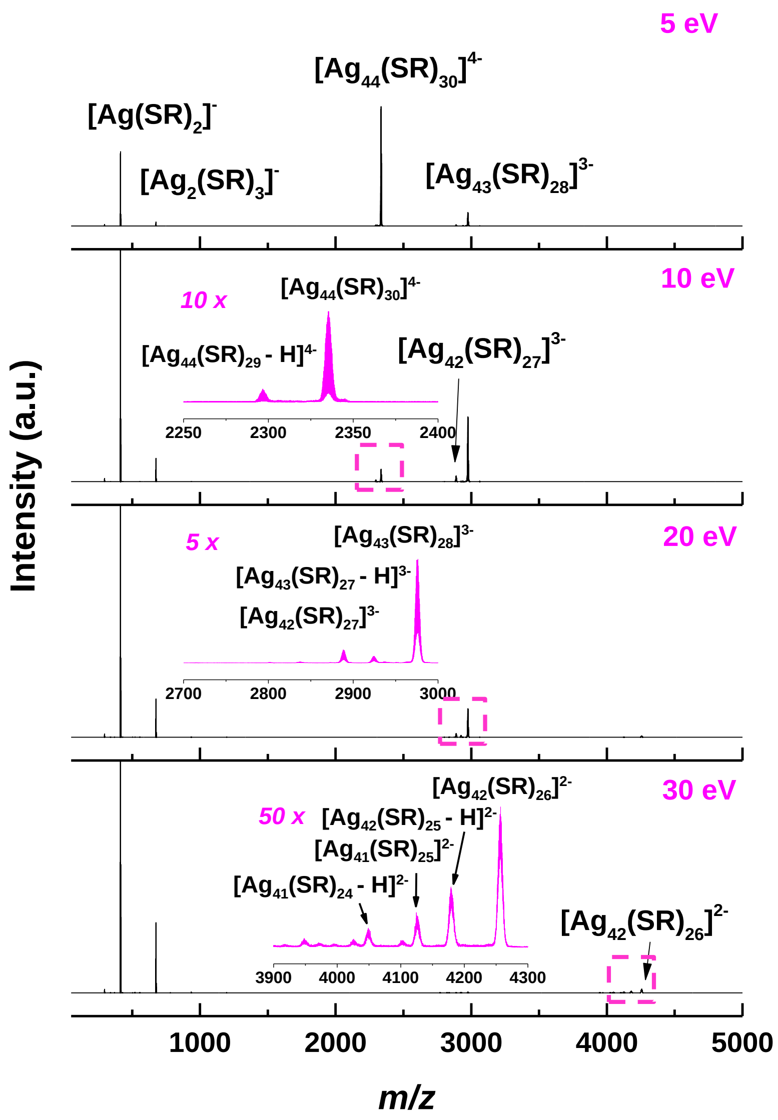

Supplementary Figure 11. Tandem mass spectra of $[Ag_{44}(SR)_{30}]^{4-}$ ion centered at *m/z* = 2335 obtained at varied collision energies. Insets are zoom-in spectra of the boxed area in corresponding panel.

Supplementary Note 2. Fragmentation habit of $[Ag_{44}(SR)_{30}]^{4-}$ NCs.

As illustrated in Supplementary Fig. 12 below, the fragmentation of $[Ag_{44}(SR)_{30}]^{4-}$ in tandem MS analysis follows a stripping-off mechanism, where the fragment ions are developed from their parent ions via successive dissociation of single negatively charged $[SR]^-$ (orange arrows), $[Ag(SR)_2]^-$ (blue arrows), and $[Ag_2(SR)_3]^-$ (purple arrows). Specifically, the 1st generation fragment cluster ions (i.e., $[Ag_{44}(SR)_{29} – H]^{4-}$, $[Ag_{43}(SR)_{28}]^{3-}$, and $[Ag_{42}(SR)_{27}]^{3-}$) are developed at low collision energy (e.g., 5 and 10 eV, Supplementary Fig. 11) by dissociation of the aforementioned single negatively charged species from parent cluster ions (i.e., $[Ag_{44}(SR)_{30}]^{4-}$). Further increasing the collision energy (e.g., 20 and 30 eV, Supplementary Fig. 11) could lead to fragmentation of the 1st generation fragment cluster ions by dissociation of identical single negatively charged species, yielding the 2nd generation fragment cluster ions (i.e., $[Ag_{43}(SR)_{27} – H]^{3-}$, $[Ag_{42}(SR)_{26}]^{2-}$, and $[Ag_{41}(SR)_{25}]^{2-}$). At an extreme collision energy (e.g., 30 eV, Supplementary Fig. 11), the 3rd generation fragment cluster ions (i.e., $[Ag_{42}(SR)_{25} – H]^{2-}$ and $[Ag_{41}(SR)_{24} – H]^{2-}$), which are most probably formed by dissociation of the aforementioned single negatively charged species from the 2nd generation fragment ions, could also be observed. It should be noted that a stepwise downgrade of net charge by 1 electron (e^-) was recorded when the generation count of fragment cluster ions was increased by 1, which is in good agreement with the proposed stripping-off mechanism by successive dissociation of single negatively charged species.

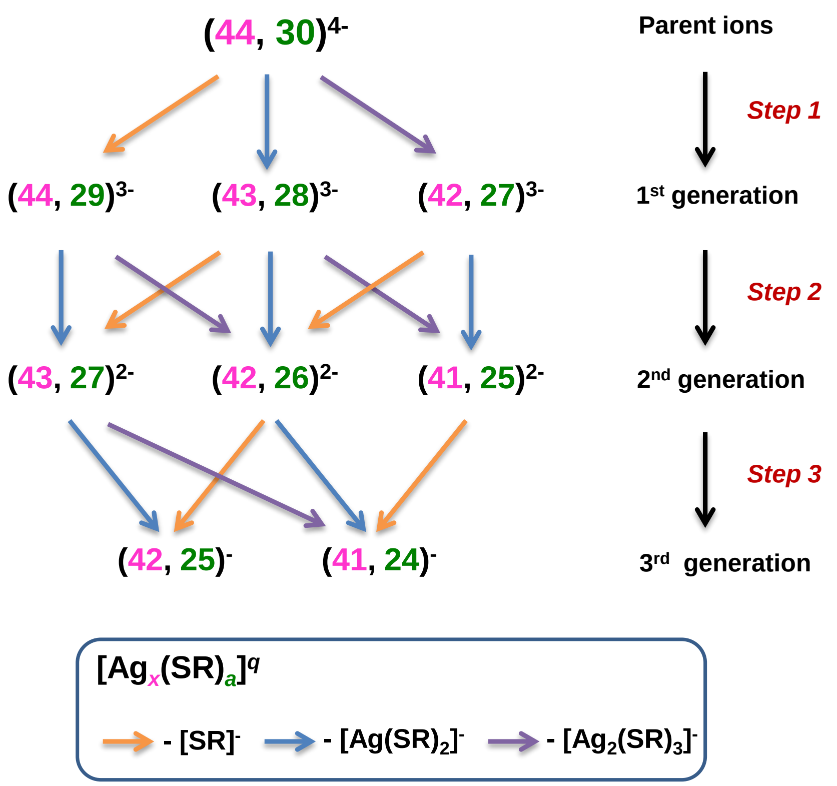

Supplementary Figure 12. Schematic illustration of fragmentation process of $[Ag_{44}(SR)_{30}]^{4-}$ ions in tandem mass spectrometry analysis, where $[Ag_x(SR)_y]^q$ is referred to as $(x, y)^q$ for clarity.

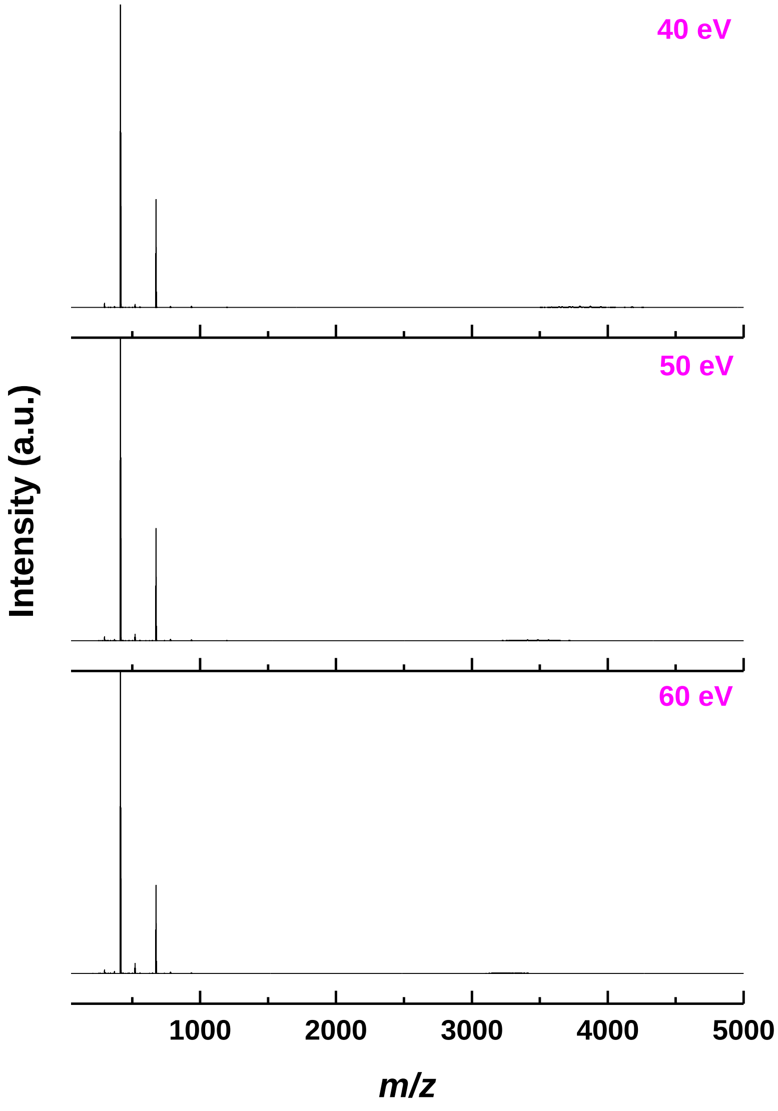

Supplementary Figure 13. Tandem mass spectra of $[Ag_{44}(SR)_{30}]^{4-}$ ion centered at *m/z* = 2335 obtained at collision energies higher than 30 eV.

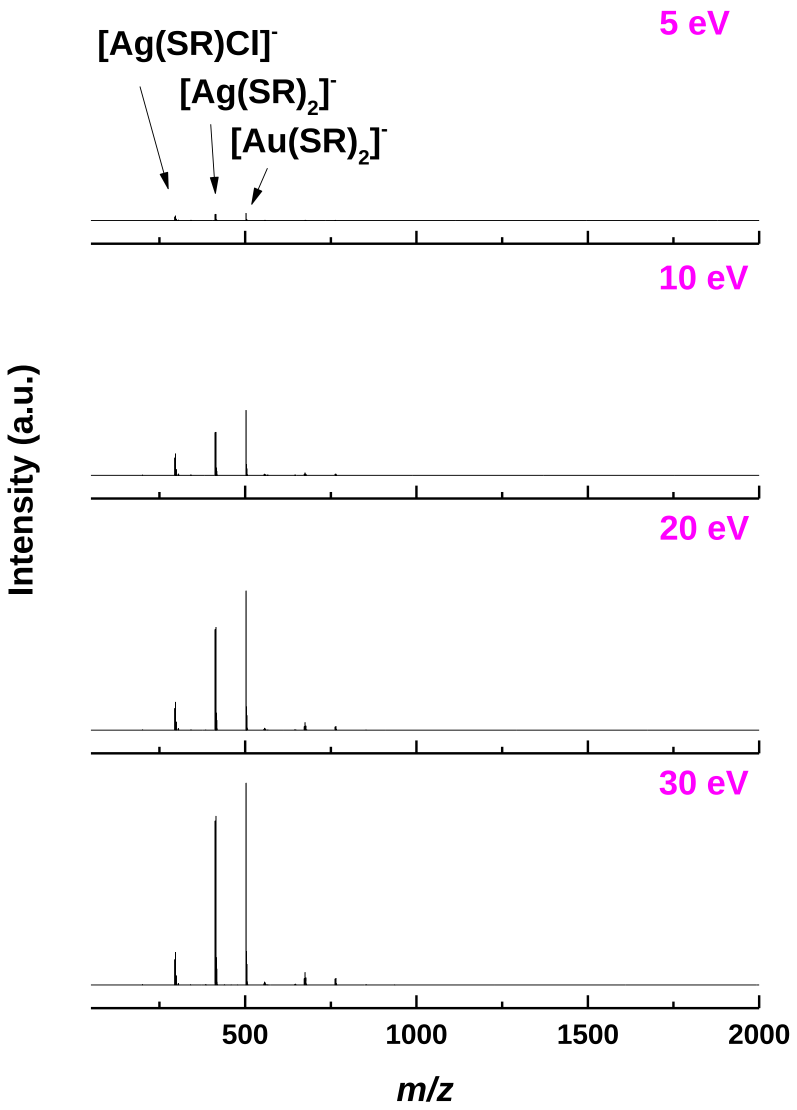

Supplementary Figure 14. Tandem mass spectra in *m/z* = 50–2000 of $[Ag_{34}Au_{10}L_{30}]^{4-}$ and $[Ag_{33}Au_{11}L_{30}]^{4-}$ (marginal) ions. Collision energies are highlighted in magenta in each panel. With increasing collision energies, the abundance of single negatively charged species $[Ag(SR)Cl]^{-}$, $[Ag(SR)_2]^{-}$, and $[Au(SR)_2]^{-}$ increases, corroborating the stripping-off fragmentation mechanism by dissociation of these species.

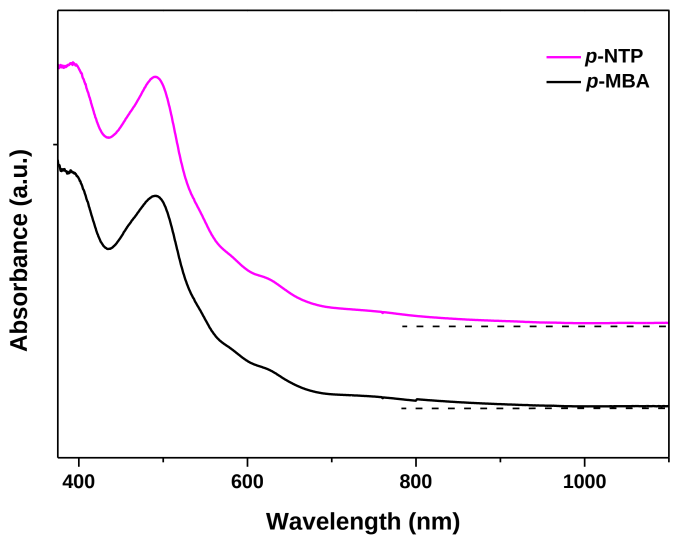

Supplementary Figure 15. Ultraviolet-visible absorption spectra of $[Ag_{44-x}Au_x(SR)_{30}]^{4-}$ nanoclusters prepared by reacting $[Ag_{44}(p\text{-MBA})_{30}]^{4-}$ with Au(I)-(*p*-NTP) (magenta line, *p*-NTP = *para*-nitrothiophenol) and Au(I)-(*p*-MBA) (black line, *p*-MBA = *para*-mercaptobenzoic acid) complexes, respectively. Zero absorbance is indicated by dotted lines.

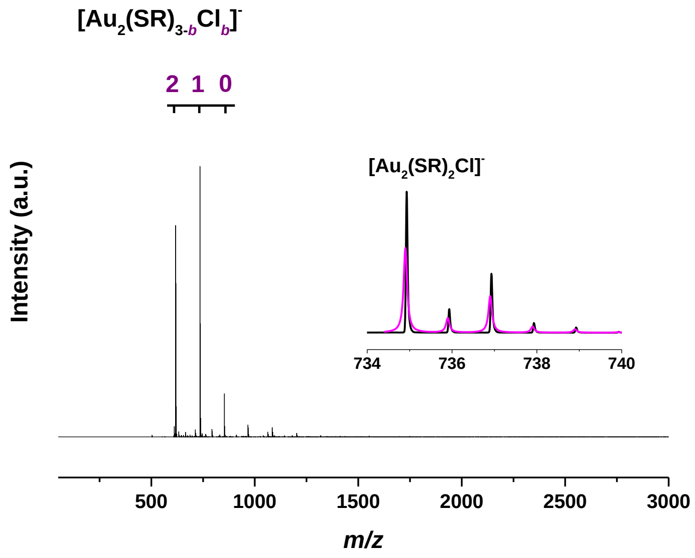

Supplementary Figure 16. Electrospray ionization mass spectrum of Au(I)-SR complexes used in surface motif exchange reaction. The inset shows experimental (black line) and simulated (magenta line) isotope patterns of $[Au_2(SR)_2Cl]^-$.

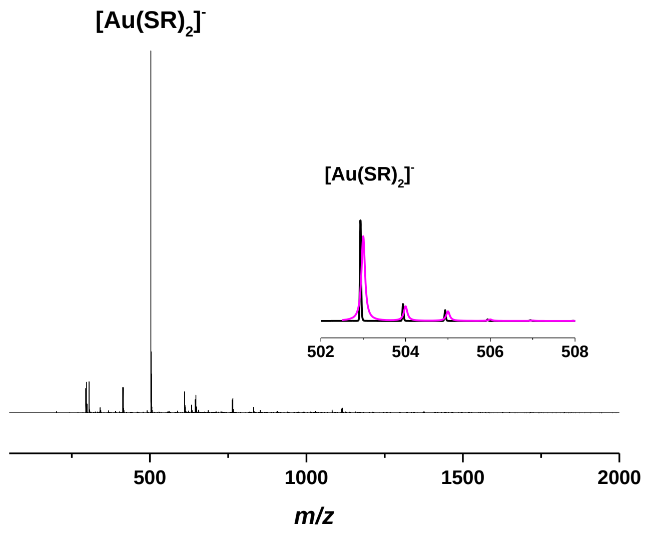

Supplementary Figure 17. Electrospray ionization mass spectrum of Au(I)-SR complex by-product observed in surface motif exchange reaction. The inset shows experimental (black line) and simulated (magenta line) isotope patterns of $[Au(SR)_2]^-$.

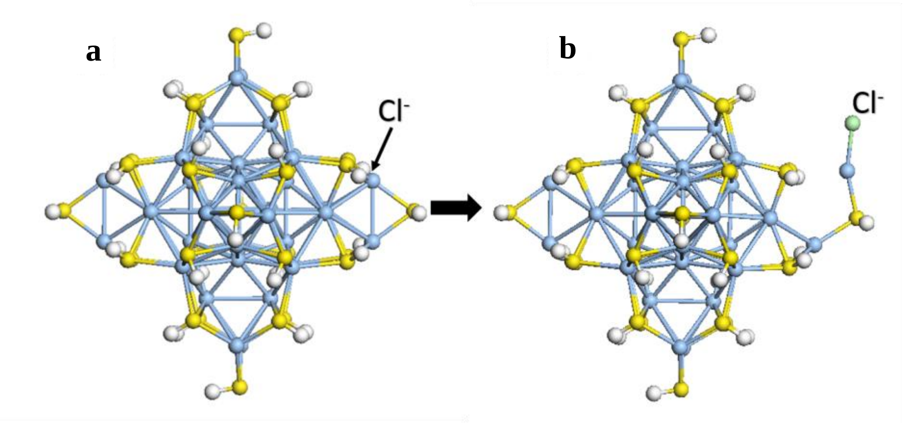

Supplementary Figure 18. **Schematic illustration of cleavage of surface Ag-S bond in $[Ag_{44}(SR)_{30}]^{4-}$ induced by Cl^-.** (**a**) Initiation of the motif exchange by Cl^- ion absorption, and (**b**) subsequent geometric relaxation and Ag-S bond cleavage induced by bonding of the Cl^- to the Ag atom. For ease of computation, the –SR is simplified as –SH.

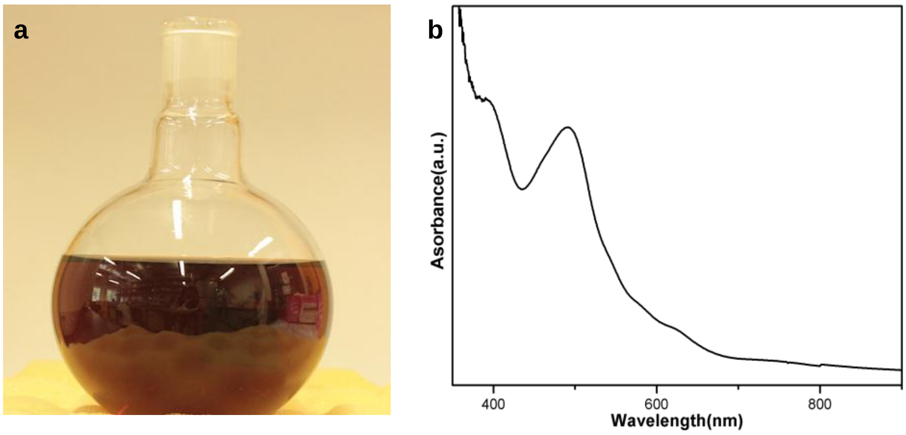

Supplementary Figure 19. (**a**) Digital image and (**b**) ultraviolet-visible absorption spectrum of $[Ag_{32}Au_{12}(SR)_{30\text{-}b}Cl_b]^{4-}$ NCs (b = 0–2) synthesized at large quantity (volume of flask = 100 mL).

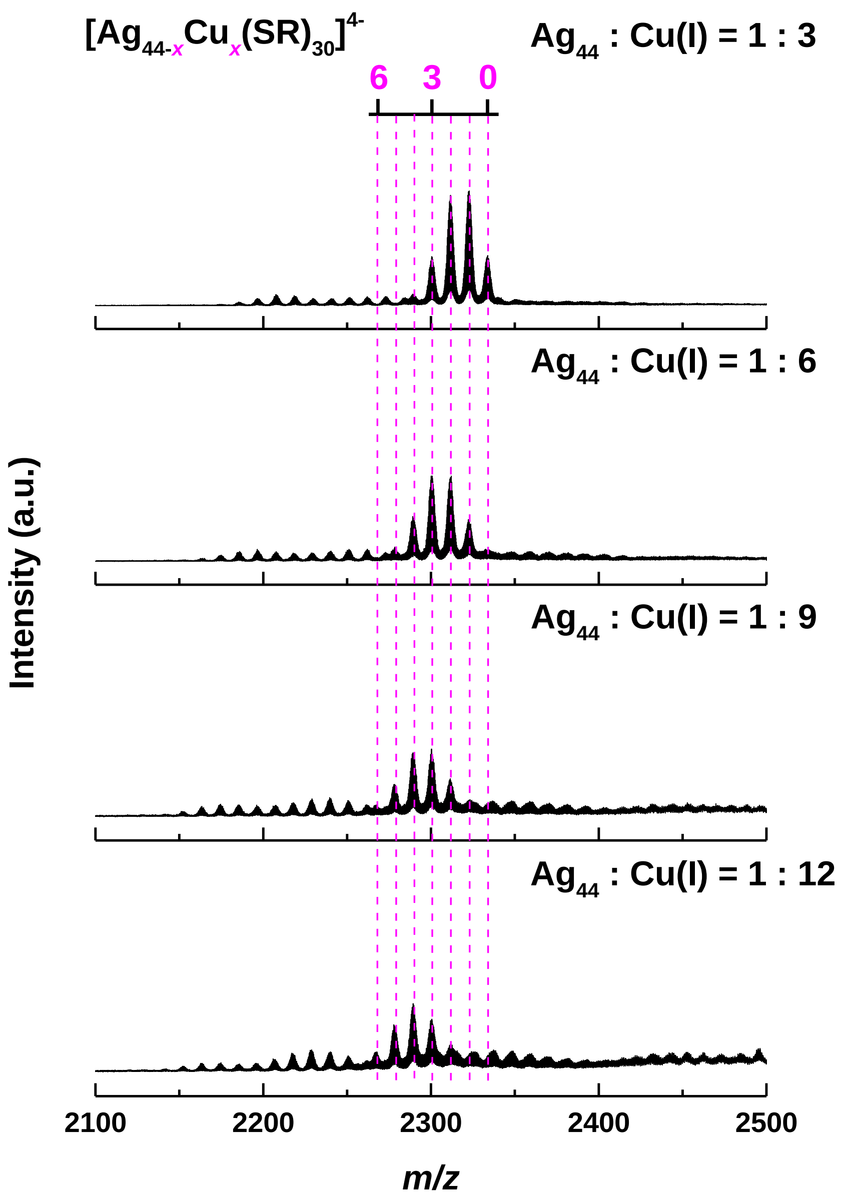

Supplementary Figure 20. Reaction of Ag_{44} nanoclusters with varied dose of Cu(I)-SR complexes. Electrospray ionization mass spectra of $[Ag_{44-x}Cu_x(SR)_{30}]^{4-}$ NCs synthesized at varied feeding ratios of Ag_{44}-to-Cu(I), $R_{Ag44/Cu(I)}$. The dotted lines indicate the number of Cu heteroatoms in each cluster.

Supplementary References

1. Yao, Q., *et al.* Counterion-assisted shaping of nanocluster supracrystals. *Angew. Chem. Int. Ed.* **54**, 184-189 (2015).

2. Desireddy, A., *et al.* Ultrastable silver nanoparticles. *Nature* **501**, 399-402 (2013).
